# Simple fractal method of assessment of histological images for application in medical diagnostics

**DOI:** 10.1186/1753-4631-4-7

**Published:** 2010-12-06

**Authors:** Wlodzimierz Klonowski, Robert Stepien, Pawel Stepien

**Affiliations:** 1 Lab. of Biosignal Analysis Fundamentals, Nalecz Institute of Biocybernetics and Biomedical Engineering Polish Academy of Sciences, Warsaw, Poland

## Abstract

We propose new method of assessment of histological images for medical diagnostics. 2-D image is preprocessed to form 1-D landscapes or 1-D signature of the image contour and then their complexity is analyzed using Higuchi's fractal dimension method. The method may have broad medical application, from choosing implant materials to differentiation between benign masses and malignant breast tumors.

## 1. Aims

Fractal and symbolic methods can be very useful for quantitative assessment and classification of images, based on analysis of experimental data such as microscopic images. Our philosophy is that to be applicable a method should preferably be really simple and easily understandable to non-specialists in the field. Presented methods are very simple and they both draw from multiple disciplines and have multidisciplinary applications.

## 2. Methods

T. Mattfeldt applied nonlinear deterministic methods from chaos theory to pattern analysis of tumor cells. He compared histological texture in 20 cases of mastopathy with 20 cases of mammary cancer. Epithelial texture plays a central role in histopathological diagnosis and grading of malignancy. T. Mattfeldt *pre-processed *microscopic 2-dimensional images of tumor cells' epithelium into 1-dimensional 'signals' (so called *'landscapes'*) and then by embedding these signals in a phase space using 'time-delay' method; he found that correlation dimension differs considerably between benign and malignant mammary gland tumors [1]. We have proposed to use a similar simple method for pre-processing of the surface's 2-D image to construct from any 2-D image two 1-D landscapes, but in the second step we use much simpler and more appropriate in this case Higuchi's fractal dimension method for analysis of the obtained landscapes. It evaluates the total 'length' *L(k) *of the curve defined by every *k*-th point and then determines the fractal dimension *D_f _*from the scaling that *L(k) *is proportional to $k^{-D_{f}}$ (cf. [2] - [3]).

R.M.Rangayyan and T.M.Nguyen used fractal analysis of contours of breast masses in mammograms to differentiate between malignant and benign tumors. They computed fractal dimension of contours of breast masses obtained from mammographic images calculated either directly from the 2-D contour or from a 1-D 'signature' derived from the contour applying either the ruler method or the box counting method [4]. Again, we propose to use Higuchi's method for analysis of '*signatures' *- the method is simpler and leads to comparable results.

Higuchi's fractal dimension, *D_f _*, is calculated directly from the data series, without embedding the data in a phase space. It is, in fact, fractal dimension of the curve representing the series, and so it is always between 1 and 2, since a simple curve has, of course, dimension equal 1 and a plane has dimension equal 2. The fractional part of *D_f _*is a measure of the series *complexity*. It should not be misled with fractal dimension of an attractor in the system's phase space.

### 2.1. Analysis of '*landscapes*' obtained from an image

A digitized image is a pattern stored as a rectangular data matrix. Grayscale images are matrices where the matrix elements can take on values from *g_min _= 0 *to *g_max _= (2^b^-1)*, where *b *denotes the number of bits (*g_max _= 255 *for *b = 8*). The rendering on a video screen is a presentation of the values from black (*0*) to white (*2^b ^- 1*). Most color images are overlays of three monochrome images.

Stepping through a gray value image length of *N *pixels and height of *M *pixels row by row we calculate the sum of the gray values in each row, ***G***_***m ***_, for *m = 1,...,M*. Normalizing the numbers by using the largest of those values, ***G***_***mmax***_, we produce the series of real numbers *NGS *_ℇ_*[0 1] *that we call *'horizontal landscape'*

(1) $$\begin{array}{cc} NGS_{m}=G_{m}/G_{mmax} & \left(m=1,...,M\right) \end{array}$$

and we call Higuchi's fractal dimension of this *NGS *series *D*_*h*_.

Similarly, stepping through the same image length of *N *pixels and height of *M *pixels column by column we calculate the sum of the gray values in each column, ***G***_***n ***_, for *n = 1,...,N*. Normalizing the numbers by using the largest of those values, ***G***_***nmax ***_, we produce the series of real numbers *NGS*_ℇ_*[0,1] *that we call '*vertical landscape*'

(2) $$\begin{array}{cc} NGS_{n}=G_{n}/G_{nmax} & \left(n=1,...,N\right) \end{array}$$

and we call Higuchi's fractal dimension of this *NGS *series *D*_*v*_.

Both landscapes are then analyzed using Higuchi's fractal dimension method. ([5,6]). Normalization in (1) and (2) is convenient but not really necessary since fractal dimension is invariant with respect to scaling of the data.

### 2.2. Analysis of '*signatures*' obtained from an image contour

Having the contour of an image specified in any rectangular coordinate system, i.e. by the set of pairs (*x_i _, y_i _*) such that pairs *i-*1, *i, i+*1 correspond to consecutive points on the contour for any *i = *1,...,I; the first point *i = *1 may be chosen arbitrary and the point *i = I+*1 coincides with the point *i = *1 i.e. the contour is a closed planar curve.

We calculate arithmetic averages, *x_0 _*and *y_0 _*of coordinates of all contour points and transform rectangular coordinates into polar ones; it is enough to calculate *r*-coordinate

(3) $$r_{i}^{2}={\left(x_{i}-x_{0}\right)}^{2}+{\left(y_{i}-y_{0}\right)}^{2}$$

The series *r_i _*is a 1-D '*signature' *of the 2-D contour and we analyze signatures of breast masses contours using Higuchi's method.

## 3. Results

**Figure 1:**
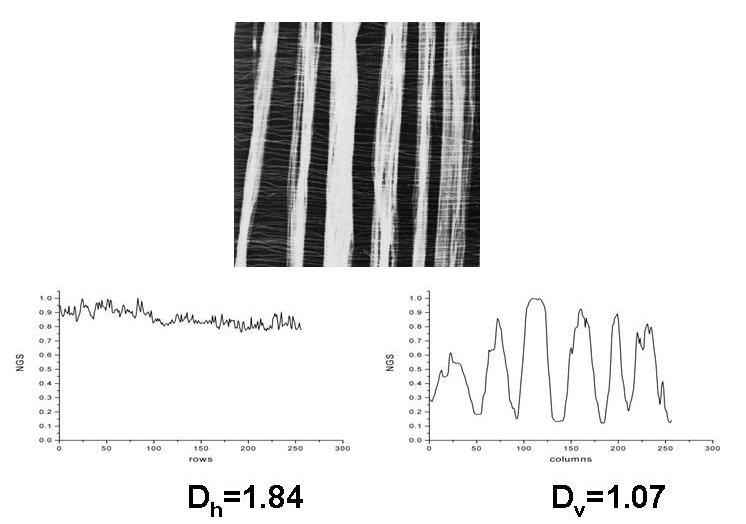
**Example of assessment of surface texture using fractal analysis of landscapes**.

**Figure 2:**
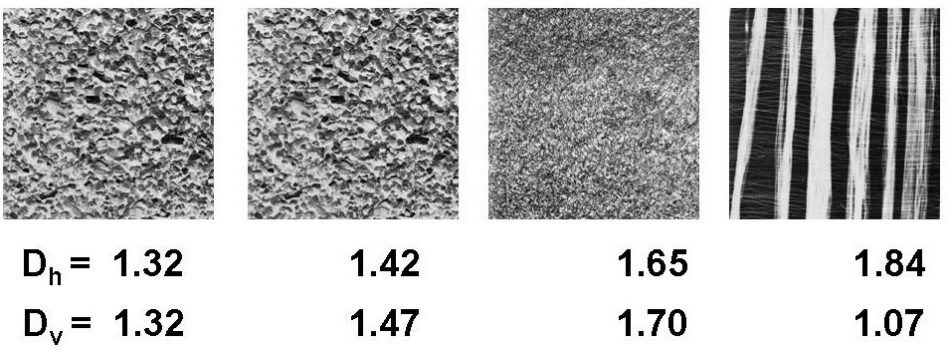
**Example of assessment of surface roughness using fractal analysis of landscapes**.

In [5] we have demonstrated that differences in fractal dimension of horizontal and vertical landscapes characterize surface texture (Figure 1) and that one may quantitatively characterize surface roughness analyzing landscapes obtained from images (Figure 2); such quantitative comparison may be used if the images are obtained using the same magnification. As examples we took surface images provided on Internet by T. Randen [7].

**Figure 3:**
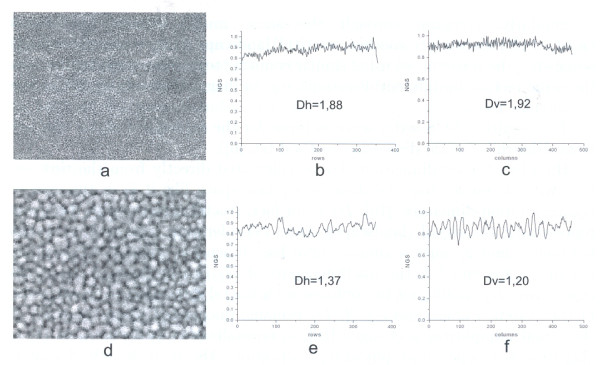
**Images of titanium surface after BSP biomimetic treatment and corresponding landscapes **- horizontal (*row by row*) and vertical (*column by column*); magnifications 700× (a-c), 3500× (d-f); fractal dimension calculated in a window of 128 points, moved in each step 1 point to the right.

**Figure 4:**
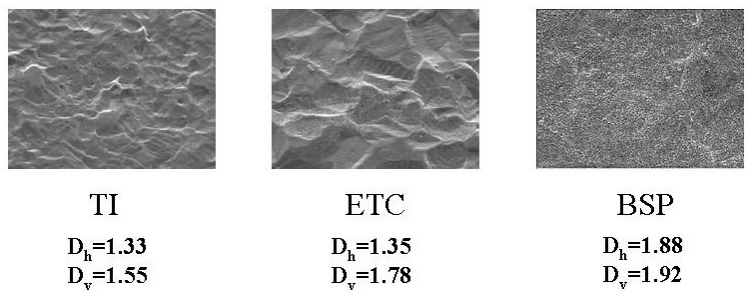
**Assessment of surface roughness of implant materials for orthopedic prostheses** - TI - titanium-coated untreated, ETC - chemically etched titanium, commercially available, BSP - surface after new biomimetic treatment; magnifications 700×.

We have used the same method for roughness assessment of implant materials based on analysis of SEM images (cf. [6], we analyzed images by C. Giordano et al. [8]). For example, we can compare quality of surfaces of implant materials for orthopedic prostheses - titanium-coated untreated and treated with different chemicals (Figure 3, 4).

Surface roughness plays an important role in cell adhesion to the surface, so quality of materials used for implants depends on their surface properties. The greater is fractal dimension the better it is as implant material. Experiment with culturing cells showed that cell adhesion is really the best for BSP-treated. surface significantly increasing cell proliferation [8]. BSP surface shows also evident multifractal properties - fractal dimension strongly decreases with magnification (Figure 3.), while for untreated titanium coated surface fractal dimension does not change with magnification; when a cell culture grows on such a surface fractal dimension decreases in comparison with that of 'naked' surface [6].

**Figure 5:**
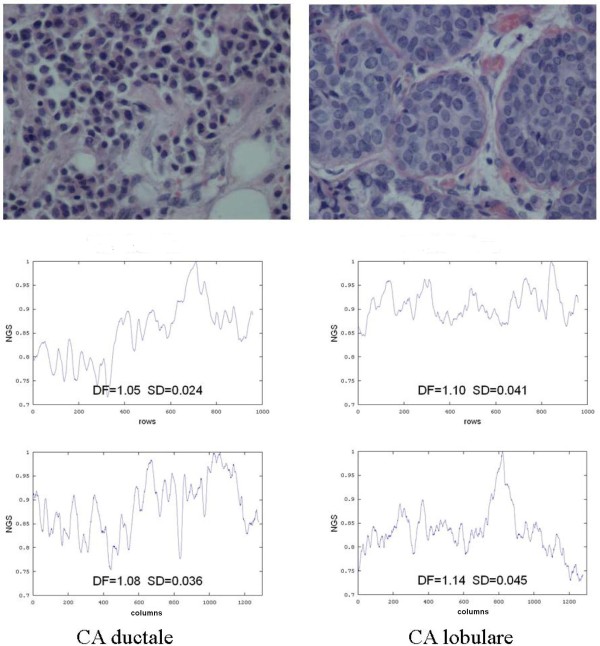
**Microscopic images of two types of breast cancer, *Carcinoma Ductale *(left) and *Carcinoma Lobulare *(right) and the landscapes of these images below (horizontal landscapes in the middle and vertical landscapes in the bottom) with their Higuchi's fractal dimensions**.

Our method may also help to distinguish between different types of cancer (Figure 5.).

**Figure 6:**
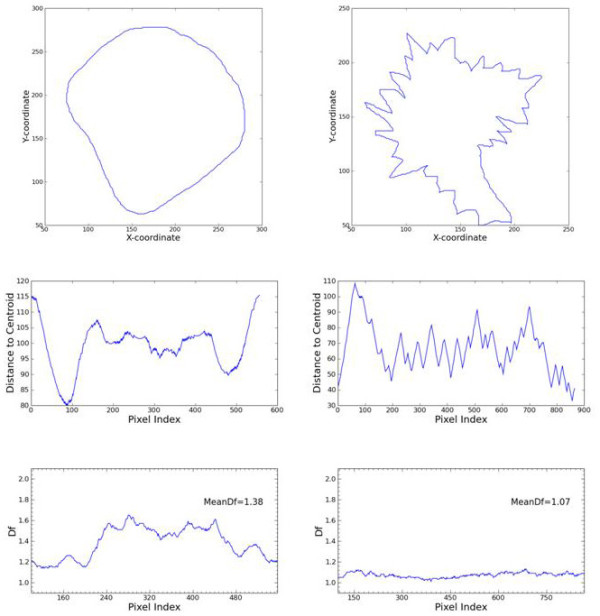
**Contours of a benign mass (upper left) and of malignant breast tumor (upper right) their signatures (cf. Eq. (3), middle row) and the signatures' Higuchi's fractal dimension (lower row)**. Signature of a malignant tumor shows lower fractal dimension than that of a benign mass.

We have also applied analysis of Higuchi's fractal dimension to the contours of breast masses (cf. [4]). Signatures of contours (cf. Eq. (2)) of benign masses show significantly higher values of Higuchi's fractal dimension than those of malignant breast tumors (Figure 6. and Table 1.). We lack numerical mammographic images of very high quality to test our method of landscapes calculated for cases of breast tumors, benign masses, cysts etc. in comparison with normal breast tissues.

**Table 1:** Higuchi's fractal dimension of the signatures of benign masses and of malignant breast tumors calculated either from the whole signature at once ('Global'), or calculated in moving window shifted in each step one element of a signature (Figure 6. c. and d.) to the right (100-elements window, kmax= 4) respectively, so giving the graphs shown in Figure 6. e. and f., and only then averaged ('Window')

	Benign		Malignant	
	**'Global'**	**'Window'**	**'Global'**	**'Window'**

	***D***_***f***_	***D***_***f***_	***D***_***f***_	***D***_***f***_

1	1,21	1,22	1,08	1,08

2	1,17	1,21	1,07	1,07

3	1,57	1,6	1,12	1,18

4	1,4	1,44	1,1	1,12

5	1,04	1,04	1,09	1,09

6	1,23	1,23	1,04	1,05

7	1,4	1,44	1,03	1,03

8	1,19	1,22	1,01	1,01

9	1,23	1,26	1,02	1,02

10	1,1	1,16	1,04	1,05

11	1,22	1,26	1,01	1,01

12	1,44	1,48	1,04	1,04

13	1,19	1,19	1,03	1,03

14	1,63	1,63	1,08	1,08

15	1,47	1,47	1,06	1,08

16	1,22	1,23	1,19	1,23

17	1,3	1,38	1,04	1,05

18	1,57	1,63	1,1	1,14

19	1,1	1,1	1,14	1,17

20	1,22	1,24	1,04	1,04

21	1,31	1,32		

22	1,36	1,37		

23	1,23	1,24		

24	1,28	1,31		

25	1,32	1,34		

26	1,41	1,41		

27	1,1	1,16		

28	1,37	1,42		

29	1,47	1,46		

30	1,22	1,26		

31	1,35	1,42		

32	1,41	1,48		

33	1,13	1,2		

34	1,23	1,27		

35	1,42	1,46		

36	1,32	1,34		

37	1,15	1,2		

***Mean D_f _***	**1,297**	**1,327**	**1,067**	**1,079**

*Standard Deviation*	0,143	0,144	0,047	0,061

While the contour of a benign breast mass (Figure 6a.) seems to be more regular than the contour of a malignant breast tumor (Figure 6b.) fractal dimension of the malignant breast tumors is lower than fractal dimension of benign breast masses (Table 1.). If the contours are blown up one may observe that these of benign masses show many small irregular than those of malignant tumors. That is why signature of a benign mass shows many small 'fluctuations' while that of a malignant tumor does not (cf. Figure 6c. and Figure 6d. respectively) so leading to differences in their fractal dimension. Problem of calculation of the length of coast-line considered by Mandelbrot is quite analogous; in fact, fractal dimension turned out to be the best characteristics that actually gives possibility to compare properties of different coast-lines [9].

We analyzed 37 cases of benign masses and 20 cases of malignant tumors. Table 1. shows that there exist significant differences in mean values of ***D***_***f ***_between signatures of benign mass contours and those of malignant tumors contours - these of benign masses are significantly higher. Also there are only small differences in *D_f _*if calculated from the whole signature at once ('Global' columns) or if calculated in a sliding window and only then averaged ('Window' columns), so our method enables really quick data analysis of the whole signature at once for check-up examinations. Standard deviations of all mean *D_f _*values are small and the ranges (*MeanD_f-bengn _±_SD_benign_*) and (*MeanD_f-malignant _±_SD_malignant_*) do not overlap. There are some outliers in each group - they would need further more detailed examination.

## Conclusions

Fractal dimension of landscapes obtained from surface images does change with the surface properties. The smoother is a surface, i.e. the smaller are its unevenness at any particular scale, the greater is fractal dimension of any landscape obtained from an image of this surface at given magnification. If a surface shows anisotropic roughness properties (texture) then fractal dimensions of its horizontal and vertical landscapes differ from one another. We demonstrated that our method may be applied for choosing better implant materials. The same method may be used in histology to help distinguish between different types of cancer.

Fractal analysis of signatures of contours of breast masses may help in differentiating between mammographic images of benign masses and malignant tumors in screening medical examinations. The method is quick - one may analyze the whole signature at once to calculate Higuchi's fractal dimension of the signature.

Our method draws from multiple disciplines and may find multidisciplinary applications. The same fractal data-processing method may be used for extraction, fusion, and visualization of multi-modal information from (nano)sensors. as well as in hybrid modeling of living organism - the method is computationally effective and may be applied in real-time.

For more detailed fractal analysis of Rangayyan's data cf. [10].

## Competing interests

The authors declare that they have no competing interests.

## Authors' contributions

The authors contributed equally to this article. All authors read and approved the final manuscript.
